# Virtual Reality Technology Reduces Pain and Anxiety in Hospitalized Pediatric Patients Undergoing Peripheral Venous Catheterization: A Randomized Controlled Trial

**DOI:** 10.3390/children13040509

**Published:** 2026-04-05

**Authors:** Jiao Yu, Qiqi Cheng, Min Luo, Huidan Yu, Suqing Wang

**Affiliations:** 1 School of Nursing, Wuhan University, Wuhan 430072, China; yujiao0212@whu.edu.cn (J.Y.); 2022283070023@whu.edu.cn (Q.C.); 2 Union Hospital, Tongji Medical College, Huazhong University of Science and Technology, No. 1277 Jiefang Avenue, Jianghan District, Wuhan 430022, China; 3 Structural Heart Disease Center, ZhongNan Hospital of Wuhan University, 169 Donghu Road, Wuchang District, Wuhan 430071, China; 4 Tongji Medical College, Huazhong University of Science and Technology, 13 Hangkong Road, Qiaokou District, Wuhan 430030, China

**Keywords:** virtual reality, peripheral venous catheterization, pain, anxiety, pediatric, inpatients

## Abstract

**Highlights:**

**What are the main findings?**

- Virtual reality distraction interventions can alleviate pain and anxiety during peripheral venous catheterization in hospitalized children.
- Virtual reality technology effectively reduces pain-related heart rate variability during peripheral venous catheterization in hospitalized children, shortens catheterization time, and enhances satisfaction with pediatric nursing procedures.
- Virtual reality is safe and reliable, demonstrating good applicability in peripheral venous puncture for hospitalized children.

**What are the implications of the main findings?**

- Improve pediatric patient experience: Effectively alleviates pain and anxiety during catheterization, stabilizes physiological indicators, and enhances comfort and satisfaction.
- Enhance clinical efficiency and safety: Reduces catheterization time, increases satisfaction with nursing procedures, and is safe and reliable for clinical application.
- Implementing virtual reality interventions in the quiet and stable environment of a hospital ward helps to enhance immersion and improve the effectiveness of the intervention.

**Abstract:**

**Objective:** To investigate the effects of virtual reality (VR) technology on pain and anxiety in hospitalized pediatric patients undergoing Peripheral Venous Catheterization. **Methods:** This study is a randomized controlled trial (RCT). Between July and December 2024, eligible pediatric inpatients aged 5–14 years from the Chegu Branch of Wuhan Union Hospital were randomly assigned to either the experimental group or the control group. The control group received routine care during peripheral venous catheterization, including health education and psychological comfort. The intervention group, in addition to routine care, used VR headsets to watch age-appropriate game videos, with each VR session lasting 10–15 min. The primary outcome measure was patient-reported pain levels, with anxiety as a key secondary outcome. Secondary outcome measures included catheterization time, heart rate, patient satisfaction with nursing procedures, and usability evaluation of the VR equipment. **Results:** A total of 80 pediatric patients were enrolled, with 40 in the VR group (mean age 8.05 ± 2.60 years) and 40 in the control group (mean age 8.63 ± 2.50 years). Generalized estimating equation (GEE) analysis showed a significant interaction effect between group and time for pain (Wald χ^2^ = 7.091, *p* = 0.029), while no significant interaction was found for anxiety (Wald χ^2^ = 0.971, *p* = 0.615). Before peripheral venous catheterization, there was no significant difference in pain and anxiety scores between the two groups of pediatric patients (*p* > 0.05). Patients in the VR group reported significantly reduced pain (β = −0.78; 95% CI, −1.40 to −0.15; *p* = 0.015) during catheterization, and overall anxiety scores were also lower in the VR group (β = −0.43; 95% CI, −0.77 to −0.08; *p* = 0.016), although the group by time interaction for anxiety was not significant. The intervention group also demonstrated a lower peak heart rate (107.67 ± 16.25 beats/min vs. 115.25 ± 29.53 beats/min; *p* = 0.047) and a shorter procedure duration [110 (100, 120) seconds vs. 120 (110, 123.5) seconds; *p* < 0.001]. Operator satisfaction with the nursing procedure was also significantly higher in the intervention group (95.0% vs. 72.5%, *p* < 0.001). **Conclusions:** VR significantly reduces pain and anxiety in hospitalized pediatric patients during peripheral venous catheterization.

## 1. Introduction

Hospitalization in itself constitutes a negative experience [1]. When afflicted by illness, patients not only endure physical suffering caused by the disease but also experience psychological and emotional stress responses such as tension, pressure, frustration, and sadness triggered by the state of being unwell [2]. Even adults may fall into varying degrees of anxiety. For children whose mental and cognitive functions are still developing, the distress brought by hospitalization is even more intense [3]. When hospitalized due to illness, children must leave familiar environments such as home and school to enter an unfamiliar setting. They constantly perceive the tension of their primary caregivers while enduring the pain and discomfort caused by medical procedures such as repeated blood draws and scheduled intravenous infusions, further exacerbating their fear and anxiety [4].

During hospitalization, peripheral venous catheterization is an essential procedure and the most widely used method for establishing vascular access [5]. Compared to single, brief venipunctures, the catheterization process is more complex and time-consuming, inevitably prolonging the child’s anticipation of pain and experience of discomfort. International studies indicate that 50% to 60% of children report significant pain and anxiety during venous puncture, with nearly 63.0% subsequently developing “needle phobia” [6,7,8], which can lead to childhood phobias, pre-treatment anxiety, and treatment resistance [9]. Distraction techniques refer to a coping strategy that involves guiding an individual’s attention toward non-painful stimuli, thereby temporarily shifting their focus away from distressing experiences such as pain or anxiety [10]. This approach has been demonstrated to effectively minimize children’s fear, anxiety, and perception of acute pain associated with venous access procedures [11]. VR technology, as an advanced non-pharmacological and non-invasive distraction intervention, uses head-mounted devices to create a fully immersive and interactive virtual environment for children [12]. This approach achieves a cognitive and emotional “transfer” of the child away from the immediate medical context, reducing attention to pain signals and the brain’s perception of pain, thereby alleviating the child’s pain and anxiety [13].

Currently, evidence-based practice internationally has gradually confirmed the positive role of VR in pediatric procedural pain management [14,15]. While international studies on this topic are abundant, they primarily focus on pediatric outpatient and emergency settings [13,16,17], with relatively insufficient research on inpatient ward environments. Hospitalized children often undergo repeated medical procedures, and the psychological stress they experience is both cumulative and context-specific, warranting in-depth exploration [18]. This perspective aligns with the broader landscape of assistive and emerging technologies designed to detect and reduce neurophysiological stress and anxiety in children [19], further supporting the rationale for implementing VR-based interventions in hospitalized pediatric populations. A recent high-quality study [20] demonstrated that the use of commercially available VR devices during peripheral venous catheterization in a pediatric emergency department did not yield significant positive effects. This finding suggests that the efficacy of VR-based distraction interventions may be moderated by multiple factors, including environmental urgency, the child’s baseline anxiety level, the age-appropriateness and immersiveness of VR content, and the adequacy of pre-procedural preparation. In fast-paced, high-pressure emergency settings, commercial VR devices may be insufficient to effectively capture children’s attention within limited preparation time. In contrast, the more controlled and predictable environment of inpatient wards may allow for better-tailored and more thoroughly prepared VR interventions, potentially enhancing their therapeutic effects.

Therefore, to address the inconsistencies in the aforementioned findings and further explore the application value of VR in inpatient pediatric care, this study employs the Attention Resource Limitation Theory [21] and the Gate Control Theory [22] as its theoretical frameworks. The Attentional Resource Limitation Theory posits that an individual’s total attentional capacity is limited. When VR experience engages a significant portion of this capacity, the cognitive resources available for processing pain are consequently reduced. The Gate Control Theory explains the underlying neural mechanism, proposing that non-noxious sensory input (such as the visual and auditory stimuli provided by VR) can activate inhibitory interneurons in the spinal cord’s dorsal horn. This activation effectively “closes the gate,” impeding the transmission of pain signals to the brain. Therefore, by delivering a highly immersive multi-sensory experience, the VR intervention is anticipated to competitively occupy the child’s attentional resources and engage the endogenous pain-inhibitory pathways, thereby achieving a reduction in the perception of pain and anxiety. By focusing on the stable setting of inpatient wards and comparing VR interventions with conventional care, this research aims to determine whether VR can reliably reduce pain and anxiety in hospitalized children. Additionally, this study will systematically evaluate the applicability, tolerability, and potential limitations of VR devices in hospitalized children, with the goal of providing empirical evidence and practical references for clinical practice.

## 2. Methods

### 2.1. Study Design and Participants

We conducted a single-center, parallel-group, randomized controlled trial (registered on 18 December 2025, at the Chinese Clinical Trial Registry; registration number: ChiCTR2500114904). This study report adheres to the Consolidated Standards of Reporting Trials (CONSORT) guidelines [23].

This study employed convenience sampling to select pediatric patients requiring peripheral venous catheterization in the pediatric ward of Union Hospital, Tongji Medical College, Huazhong University of Science and Technology, from July to December 2024. Inclusion criteria: (a) Hospitalized children aged 5–14 years; (b) Children with normal comprehension and communication abilities who actively participated; and (c) Voluntary participation in the trial. Exclusion criteria: (a) Children with visual or auditory impairments; (b) Children with psychiatric history, severe cardiovascular disease, or other physical illnesses; (c) Children unable to fully complete questionnaires with nursing assistance; (d) Children who had taken analgesics within the past week; and (e) Uncooperative children, defined as those who refused to follow verbal instructions during the pre-procedure preparation or expressed unwillingness to undergo the catheterization despite caregiver reassurance.

The sample size calculation formula for comparing two-sample means in experimental studies [24] is:

(1) $$n_{1}=n_{2}=2{\left(\frac{u_{\alpha }+u_{\beta }}{\delta /\sigma }\right)}^{2}+\frac{1}{4}u_{\alpha }^{2}$$

where *n*_1_ and *n*_2_ represent the sample sizes for the two groups, *n_2_* represent the sample sizes for the two samples, and *u_α_* and *u_β_* denote the u-values corresponding to the significance level *α* and Type II error probability *β*, respectively. σ is the estimated standard deviation of the two populations (replaced by the sample standard deviation *S)*, and *δ* is the difference between the means of the two continuous variables. Based on relevant literature [24], *S* = 8.81, *δ* = 6.15, *α* = 0.05, and *u_α_* = 1.96 were selected; With *β* = 0.20, *u_β_* = 0.8416. Substituting these values yields *n_1_* = *n_2_* = 34 cases. Considering an approximate 15% sample attrition rate and non-response bias, the experimental and control groups each comprise 40 cases.

### 2.2. Randomization and Blinding

This study employed simple randomization with a 1:1 allocation ratio. The randomization sequence was generated by an independent statistician using computer-generated random numbers. Allocation concealment was ensured through the use of sequentially numbered, sealed, opaque envelopes to maintain the unpredictability of the assignment process. The enrolling researcher, who was not involved in sequence generation, opened the next envelope to assign eligible participants to either the intervention group or the control group.

However, due to the readily identifiable nature of the intervention (the use of VR devices), it was not feasible to blind participants, researchers, or healthcare professionals. To minimize potential bias, data analysts remained blinded to group allocation throughout the study.

The unblinding plan was as follows: unblinding would occur after the completion of the study and final statistical analysis of the data; emergency unblinding would be implemented if a child experienced serious adverse events during the study.

### 2.3. Interventions

#### 2.3.1. Establishment of the Research Team

The research team comprised one ward nurse manager, one senior nurse practitioner, one attending physician, and one nursing graduate student. The nurse manager reviewed the VR content design and supervised implementation, while the senior nurse practitioner strictly adhered to peripheral venous catheterization standards when performing the procedure and corresponding health education for both groups of pediatric patients. The nursing graduate student monitored the heart rate of both groups of pediatric patients using a finger pulse oximeter (Yuwell YX306, manufactured by Jiangsu Yuwell Medical Equipment & Supply Co., Ltd., Jiangsu, China) during the procedure and collected data on pain, anxiety, satisfaction scores, catheterization time, and VR applicability assessment forms. The attending physician monitored the patients’ reactions during the catheterization procedure and immediately addressed any adverse reactions. Adverse events, including but not limited to dizziness, nausea, eye fatigue, and any signs of distress, were actively monitored and recorded by the attending physician and nursing graduate student for all participants throughout the procedure. The intervention was to be discontinued immediately in the event of any serious adverse event.

#### 2.3.2. Development of Intervention Protocol

Literature reviews on VR applications for pediatric peripheral venous catheterization were conducted domestically and internationally. Analysis of pain and anxiety levels during catheterization, along with corresponding physiological changes, informed the initial intervention protocol developed through repeated discussions by the research team. A pilot study was conducted with 15 eligible patients in each group (control and observation). The final intervention protocol was refined based on issues identified during the pilot study. The intervention plan details are as follows: (1) Timing: After the baseline survey, the experimental group begins VR intervention upon entering the pediatric procedure room and continues it throughout the standard care procedures until the conclusion of the catheterization procedure. (2) Implementer: The same senior nurse performed peripheral venous catheterization and health education for both groups. Family members were present but did not participate in the intervention. (3) Duration: The VR intervention for the experimental group lasted from 5 min before the catheterization procedure until its completion, with the entire process taking 10–15 min; (4) Game Video Content: Five suitable 360° video contents were selected from the PICO neo3 software store after review by the research team and added to the favorites (Table 1). After review and consent by the patients’ families, the research nurse assisted in playing the videos.

#### 2.3.3. Determining Research Tools

Based on the integration of research requirements, literature analysis, and market survey results, the PICO Neo3 VR all-in-one headset was ultimately selected as the intervention tool for the experimental group due to its suitability for playing virtual videos. This study utilizes a fully enclosed virtual headset that continuously captures environmental features through four wide-angle cameras mounted on the headset. Built-in speakers on both sides deliver spatial audio for enhanced immersion, while foam padding conforms to the face and adjustable straps ensure comfort for extended wear. When pediatric patients wear the VR headset to view virtual content, the three-dimensional images dynamically adjust in real-time with minor head movements. This design maintains immersion while preventing significant head swivel that could interfere with medical staff operations.

#### 2.3.4. Implementation of Intervention Methods

##### Intervention Group

**Figure 1:**
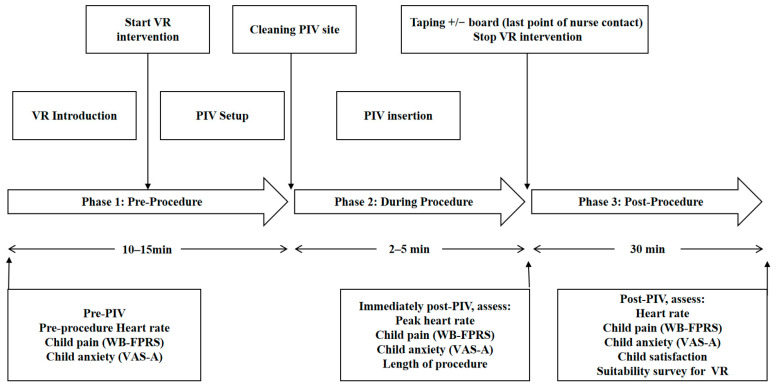
Study flow diagram.

Upon entering the pediatric procedure room, children in the experimental group received routine care. A researcher then demonstrated the virtual reality equipment, explaining its operation in simple, easy-to-understand language. The researcher assisted the child in wearing the headset and invited them to try it for 5 min. After opening the PICO Store favorites interface, children of different ages and genders selected videos based on their preferences. Following the 5 min trial, the researcher notified the nurse to begin catheterization. During this procedure, the child continued using the VR headset until completion. The researcher then assisted in removing the headset, inquired about the child’s experience, and guided them through completing various assessment forms. Throughout the virtual reality experience, researchers remained present to observe the children’s reactions (Figure 1).

##### Control Group

A senior nurse performed peripheral venous catheterization and routine care for enrolled patients. This included pre-procedure explanations to patients and families regarding the purpose of the catheterization, assessment of the insertion site, and detailed instructions on the procedure and related precautions. They covered daily catheter maintenance, preventing catheter dislodgement, fixation methods, daily life considerations, and potential catheter-related complications such as bleeding, post-removal needle site care, etc. Prior to the procedure, the nurse performing the placement encouraged and reassured the child to alleviate anxiety. Family members were also permitted to provide comfort, such as through hugs.

### 2.4. Measurements

#### 2.4.1. Pain Level

The primary outcome was pain, assessed using the Wong–Baker Faces Pain Rating Scale (WB-FPRS) in both groups of pediatric patients: 10 min prior to peripheral venous catheterization (T0), at the conclusion of catheterization (T1), and 30 min post-catheterization (T2). Developed by Wong et al. [25], this scale comprises six facial expressions ranging from smiling to sadness to crying. Scores ranged from 0 to 10, representing no pain, little pain, mild pain, noticeable pain, severe pain, and intense pain, respectively. Each score level corresponded to a specific facial expression. Children selected one expression to represent their pain intensity at the time of scoring. This scale is suitable for assessing acute pain in children [26].

#### 2.4.2. Anxiety Assessment

Anxiety was assessed as a key secondary outcome using the Visual Analog Scale for Anxiety (VAS-A) at 10 min before peripheral venous catheterization (T0), at the completion of catheterization (T1), and 30 min after catheterization (T2). The scale ranges from 0 to 10. The leftmost score of 0 indicates no anxiety, while the rightmost score of 10 represents maximum anxiety. Children selected one expression to represent their pain intensity at the time of scoring. All children self-rated their pain independently, with the researcher providing standardized instructions. Caregiver assistance was permitted only if the child required help understanding the scale. This scale has demonstrated good reliability and validity for effectively measuring subjective anxiety [27].

#### 2.4.3. Comparison of Pediatric Patients’ Heart Rate, Catheterization Duration, and Procedure Satisfaction

From 10 min before peripheral venous catheterization to 30 min after its completion, heart rate and oxygen saturation were continuously monitored using a finger-clip pulse oximeter and recorded by the researchers. The data collected included heart rate 10 min before venipuncture (T0), peak heart rate during catheterization (Tmax), and heart rate 30 min after catheterization (T2). A stopwatch was used to record the time from disinfection to completion of peripheral venous catheter fixation. Patient satisfaction was assessed using a 5-point Likert scale, ranging from “completely dissatisfied” (1 point) to “very satisfied” (5 points). The satisfaction percentage (%) was calculated as (number of “very satisfied” + “somewhat satisfied” cases)/total cases × 100%.

#### 2.4.4. Suitability of VR Goggles

Factors such as the patient’s educational background, age, level of exposure to electronic devices, and tolerance for external interventions may all influence the suitability of virtual reality. To obtain authentic feedback on VR interventions, our research team conducted a systematic review of relevant literature and based on observations from prior trials, refined five questions through group discussions to facilitate a basic assessment of virtual reality’s suitability.

### 2.5. Data Collection

Prior to intervention, both groups of pediatric patients were required to provide basic information such as age and diagnosis for assessment. We provided paper-based questionnaires to the patients, which took approximately 10 min to complete. If caregivers could not recall specific medical history details, they could leave those sections blank; we would later retrieve necessary information from the medical record system. To minimize bias in outcome assessment, all subjective scale administrations were conducted by a dedicated outcome assessor who was not involved in VR delivery. The assessor used standardized, scripted instructions for each scale to ensure consistency across participants and groups. The assessor remained blinded to group allocation whenever feasible, although complete blinding was limited by the observable nature of VR use. Peripheral venous catheterization procedures and health education for both groups were performed by the same senior nurse. Physiological data and procedure duration were recorded by the outcome assessor using standardized forms. All data were double-checked and entered to ensure completeness and accuracy.

### 2.6. Data Analysis

In formal studies, data were entered and verified by two individuals before statistical analysis was performed using SPSS 27.0 software. Quantitative data were first assessed for normality through the Shapiro–Wilk test. Quantitative data conforming to normal distribution were expressed as mean ± standard deviation (mean ± SD), with intergroup comparisons performed using the independent samples *t*-test. Non-normally distributed quantitative data were expressed as median (25th percentile, 75th percentile), i.e., M (*P*_25_, *P*_75_), with intergroup comparisons using the Mann–Whitney U test. Categorical data were compared between groups using the chi-square test or Fisher’s exact probability method. Pain and anxiety scores at different intervention time points were analyzed using Generalized Estimating Equations (GEE), with grouping, time points, and the interaction between grouping and time points serving as explanatory variables. For the GEE models, a Gaussian distribution with an identity link function was assumed for both pain and anxiety scores, given their approximately continuous nature. An unstructured working correlation matrix was specified to account for within-subject correlations across time points without imposing a predefined pattern. Robust standard errors were used to obtain valid inference even if the working correlation structure was misspecified. No missing data occurred for pain or anxiety outcomes at any time point, as all participants completed all assessments. For normally distributed data, Cohen’s d was used to estimate the effect of the VR intervention, while for skewed data, the Rank-biserial r effect size was employed. For normally distributed data, Cohen’s d was used to estimate the effect of the VR intervention, calculated as the difference between group means divided by the pooled standard deviation. For skewed data, the Rank-biserial r effect size was employed, derived from the Mann–Whitney U test statistic to quantify the magnitude of the group difference. A *p*-value < 0.05 was considered statistically significant. Consistent with the registered trial protocol (ChiCTR2500114904), pain was pre-specified as the primary outcome, with the primary contrast defined as the between-group difference at T1 (immediately after catheterization). Anxiety and all other outcomes were considered secondary or exploratory. No multiplicity adjustment was applied, as the confirmatory analysis was limited to a single primary endpoint at a single primary time point.

### 2.7. Ethical Considerations

This study has been approved by the Ethics Committee of Union Hospital, Tongji Medical College, Huazhong University of Science and Technology (No. 2024-0590-01). All patients and their caregivers voluntarily participated, and informed consent was obtained through parental signatures. The study protocol and ethical approval were completed prior to participant enrollment, and data analysis was conducted before registration. The trial was registered with the Chinese Clinical Trial Registry (ChiCTR2500114904) on 18 December 2025.

## 3. Results

### 3.1. Participant Characteristics

**Figure 2:**
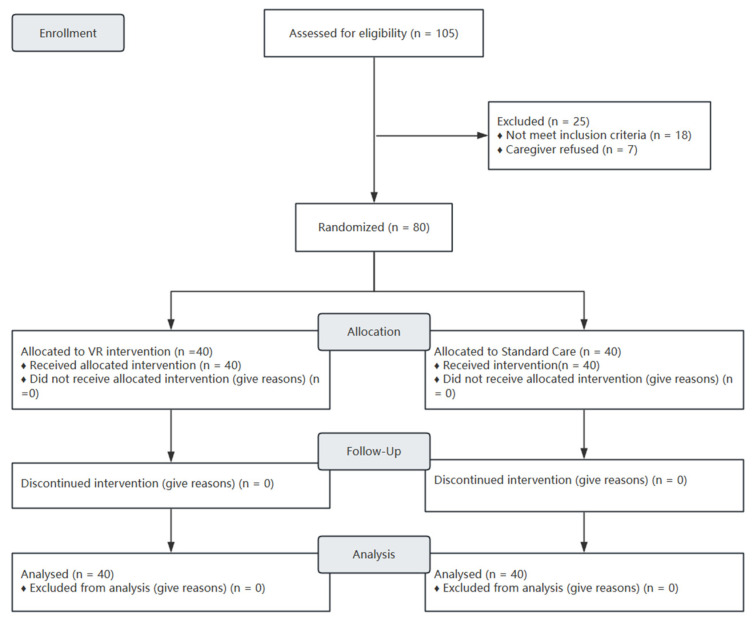
CONSORT diagram.

A total of 105 children were assessed for eligibility. Of these, 25 were excluded (18 did not meet inclusion criteria, 5 declined to participate, and 2 were excluded due to uncooperative behavior as defined in the exclusion criteria). The remaining 80 participants were randomized (40 per group) and included in the final analyses (See Figure 2 CONSORT diagram). Comparison of general data between the two groups showed no statistically significant differences (*p* > 0.05), as shown in Table 2. Thirty-one (38.75%) were female, and 53 (66.25%) had respiratory system disease. The mean age was 8.05 ± 2.60 years in the intervention group and 8.63 ± 2.50 years in the control group.

### 3.2. Pain and Anxiety

At baseline (T0), there were no significant differences in pain and anxiety scores between the two groups, indicating comparability between the groups (*p* > 0.05). Post-procedure and 30 min post-procedure pain scores were significantly lower in the experimental group compared to the control group (*p* < 0.05), as shown in Table 3. According to GEE model, the main effect of the grouping factor for pediatric pain scores was significant (*p* < 0.05), the main effect of measurement time points was significant (*p* < 0.05), and the interaction effect between grouping and measurement time was also significant (*p* < 0.05) (Table 4).

For anxiety scores, post-procedure and 30 min post-procedure pain scores were significantly lower in the experimental group compared to the control group (*p* < 0.05) (Table 3). According to the GEE model (Table 5), the main effect of the grouping factor was significant (*p* = 0.016), indicating that overall anxiety scores were lower in the VR group across time points. The main effect of measurement time was also significant (*p* < 0.001), reflecting a natural decline in anxiety over time in both groups. However, the interaction effect between grouping and measurement time was not significant (*p* = 0.700), suggesting that VR did not significantly alter the trajectory of anxiety over time compared to the control group.

### 3.3. Heart Rate and Catheterization Duration

There was no statistically significant difference in heart rate between the two groups before the procedure or 30 min after the procedure (*p* > 0.05). During the procedure, the maximum heart rate in the experimental group was significantly lower than that in the control group, with a statistically significant difference (*p* < 0.05). The procedure duration was significantly shorter in the experimental group than in the control group (*p* < 0.05), as shown in Table 3.

### 3.4. Catheterization Satisfaction

The overall satisfaction rate was significantly higher in the intervention group than in the control group (95.0% vs. 72.5%, *p* < 0.001) (Table 6).

### 3.5. VR Headset Suitability

Regarding VR headset comfort, 95% of children reported feeling comfortable; concerning fatigue, 90% reported no eye fatigue whatsoever; for overall VR headset usage ratings, 90% of children scored 8 points or higher (with 60% scoring 10 points, the maximum rating); Only 5% of patients reported mild dizziness. Regarding willingness to wear the goggles, 95% of patients expressed willingness to wear the VR goggles again for treatment, as shown in Table 7.

## 4. Discussion

Main findings: Postprocedural reports indicated that VR reduced pain scores and was associated with lower overall anxiety scores in children receiving peripheral intravenous catheterization (*p* < 0.05). This finding is consistent with the conclusions of most previous studies [7,28,29], further confirming the positive role of VR intervention in alleviating procedural pain and anxiety in pediatric patients. GEE analysis further revealed that the mechanisms by which VR affects pain and anxiety differ (Table 4 and Table 5). A significant time-by-group interaction effect was observed for pain scores (β = 0.775, Wald χ^2^ = 5.897, *p* = 0.015), indicating that VR alters the trajectory of pain over time. In contrast, no significant interaction effect was found for anxiety scores (β = 0.250, Wald χ^2^ = 0.148, *p* = 0.700), indicating that the reduction in anxiety over time followed a similar pattern in both groups, and VR did not significantly alter the trajectory of anxiety. This distinction is crucial: while VR effectively buffers the immediate pain experience by modifying its temporal course, its impact on anxiety appears to operate through a main effect—lowering overall levels—rather than by changing the natural decline of anxiety over time. From a theoretical perspective, these findings provide empirical support for the Gate Control Theory and the Attention Resource Limitation Theory. According to Gate Control Theory [22], VR provides rich visual and auditory stimuli that activate large-diameter nerve fibers, effectively “closing the gate” on pain signal transmission at the spinal cord level. The significant between-group differences and interaction effects observed in pain scores corroborate this mechanism. VR, as a “top-down” cognitive intervention, indeed alters the pain perception process [30]. The absence of an interaction effect for anxiety can be explained by Attention Resource Limitation Theory [21]: children have limited cognitive resources. Although VR can reduce absolute anxiety levels by occupying attention, it cannot override the inherent tendency for anxiety to naturally subside over time, which explains why the trajectory of anxiety reduction did not differ between groups. In other words, VR has a more direct effect on acute pain, whereas its effect on anticipatory and cognitively constructed emotional anxiety is largely superimposed upon the natural recovery process. However, compared to the negative findings of Samina Ali et al. [20] conducted in an emergency department setting, the positive results of this study may benefit from environmental advantages. Attention Resource Limitation Theory posits that a stable environment minimizes competition for cognitive resources from external stimuli, allowing VR to achieve attentional dominance. In this study, the intervention was conducted in a quiet, dedicated preparation room, providing children sufficient time to familiarize themselves with the equipment and engage with the virtual scenario—conditions conducive to VR’s effectiveness. In contrast, the chaotic and unpredictable environment of the emergency department continuously distracts children’s attention, preventing VR from effectively occupying their cognitive resources and thereby diluting the intervention effects [31]. This suggests that the clinical efficacy of VR results from the interaction between theoretical mechanisms and environmental conditions.

Pain, as a multidimensional experience, directly triggers physiological stress responses, such as changes in heart rate and blood oxygen saturation [32,33]. This study observed that the maximum heart rate of children in the VR intervention group during catheterization was significantly lower than that of the control group (*p* < 0.05). This finding not only aligns with the report by Eda Orhan et al. [34] but may also reveal the physiological benefits of VR intervention from a deeper level. The reduction in heart rate may not be solely attributed to diminished pain perception; it may signify a mitigation of the overall stress response system. When VR successfully diverts attention, the overactivation of the hypothalamic–pituitary–adrenal axis may be suppressed, leading to a decrease in sympathetic nervous system excitability, which manifests as stabilized heart rate. This improvement in physiological state, combined with the alleviation of psychological anxiety, works synergistically to create better conditions for subsequent procedures [35].

In line with this logic, this study found that the average catheterization time in the VR intervention group was significantly shorter than that in the control group (*p* < 0.05), and the children’s satisfaction with nursing procedures was higher (*p* < 0.05), which echoes the findings of Cho Lee Wong et al. [36]. The reduction in procedure time is directly attributable to the children’s higher cooperation and reduced resistance due to decreased pain and anxiety. Behind this, there may also be a more subtle interactive mechanism: when the children are calmer, the urgency and confrontational pressure faced by the operating nurses are also reduced, potentially allowing them to perform the procedure more calmly and precisely, thereby indirectly improving operational efficiency. This virtuous cycle of positive interaction between healthcare providers and patients not only enhances the efficiency and experience of a single procedure but also holds profound long-term significance for improving doctor-patient relationships, reducing medical fear, and enhancing children’s future compliance with medical care.

In terms of applicability assessment, the VR goggles demonstrated good clinical suitability in this study, which is consistent with the conclusions of Tychsen et al. [37]. Minor eye fatigue reported by a few children may be related to visual stimulation parameters of the video content, such as color saturation, contrast, or dynamic frequency. A systematic review [38] on the safety of virtual reality technology use in children under 14 years of age indicated that VR may cause mild cybersickness, and for children with amblyopia, VR use may lead to diplopia. Therefore, it is recommended to conduct a pre-assessment of the safety and suitability for pediatric patients before implementing VR intervention. The high level of interest in VR content (90% found it effective, 95% willing to use it again) demonstrates its exceptional appeal and acceptability. However, individual cases also exposed the limitations of existing devices: one child was indifferent to using VR goggles because anxiety about potential pain during treatment prevented full immersion in the game state; another child refused because not being able to see the surrounding environment made them feel more afraid; one child rated the goggles as relatively uncomfortable because they tended to slip during the procedure, affecting video viewing. This sharply highlights that current VR devices are primarily designed for adults and do not adequately consider the diverse needs of pediatric patients in terms of ergonomics (e.g., head circumference, nose bridge fit) and experience modes (e.g., whether environmental transparency is provided). Therefore, developing or adapting pediatric-specific devices suitable for different age groups and capable of flexibly adjusting immersion levels (e.g., mixed reality modes) is a key direction for future technological translation.

In summary, VR technology, through multi-dimensional mechanisms of attention capture and diversion, can effectively alleviate pain and anxiety during venous catheterization in hospitalized children in suitable environments, leading to a chain of benefits such as physiological improvement and increased operational efficiency. Due to technological revolutions and competitive marketing, VR has become more convenient and widely accessible [39]. VR is a fixed asset, as a single set of VR equipment can be reused multiple times across multiple patients. Additionally, VR hardware is easy to store and maintain. By replacing variable costs with fixed costs, virtual reality has profound implications for reducing healthcare costs [40]. It is recommended that this technology be systematically promoted and applied in pediatric inpatient wards, provided that environmental suitability is fully considered.

## 5. Limitations

Due to the inherent characteristics of VR technology, blinding of nurses and researchers was not feasible in this study, which represents a methodological limitation. The relatively narrow score range of the scale used in this study resulted in a floor effect at baseline (T0): the median pain score was 0 in both groups, the median anxiety score was 5 in both groups, and within-group variability was limited. This floor effect for pain scores at baseline is consistent with the fact that children had not yet undergone the painful procedure at the time of measurement, leaving little room for downward change in pain scores over time. Consequently, this baseline clustering phenomenon reduced the sensitivity to detect intervention effects—even if the VR intervention was truly effective, statistically significant differences would be difficult to detect. Therefore, when interpreting the between-group differences observed in this study, this limitation inherent to the measurement tool must be considered. Adverse events were actively monitored but were not pre-specified using a standardized assessment tool, which represents a limitation in harm reporting. In addition, the VR intervention condition involved a 5 min familiarization period, individualized content selection, and continuous presence of a researcher during the procedure, which constituted additional attention and interaction beyond what the control group received. This non-specific attention effect may have contributed to the observed reductions in anxiety and pain independently of the immersive VR experience itself, and this possibility should be considered when interpreting the findings. Future studies should consider enrolling populations with more pronounced baseline symptoms or adopting scales with higher sensitivity and wider score ranges to validate the intervention effects. Additionally, subjective scales may be subject to reporting bias. Additionally, there are several other shortcomings in the research: all participants were recruited from a single ward, and the limited sample size may affect the stability of the results, meaning that conclusions should be interpreted with caution when generalizing. The subjective scales used in the study may be influenced by reporting bias. Meanwhile, while the standardized VR device models and video content helped control experimental conditions, they may also restrict the applicability of the findings across different devices or intervention contents. Furthermore, as no long-term follow-up was conducted, the sustained effects of VR intervention remain unclear. Future research should involve multicenter, large-sample randomized controlled trials, incorporate diverse technical conditions and intervention protocols, and integrate objective measures with long-term follow-up to further validate the effectiveness and practical application value of VR technology.

## 6. Conclusions

In this RCT of VR intervention for hospitalized children undergoing peripheral venous catheterization, significant improvements were observed in pain, procedure duration, and patient satisfaction with the nursing procedure following VR intervention, and overall anxiety scores were lower in the VR group, although the trajectory of anxiety reduction did not differ significantly between groups. Results also indicated that virtual reality technology demonstrated good tolerability among pediatric patients, with high willingness to use it. Given that VR is becoming increasingly affordable and accessible, it holds promise for alleviating pain and anxiety associated with peripheral venous catheterization during pediatric hospitalization, thereby significantly enhancing the healthcare experience for pediatric patients.

## Figures and Tables

**Table 1:** VR Headset Video Content.

Category	Scene Name	Suitable Age (Years)	Duration	Content Introduction
Nature Real Scene	VR Tour of Chimelong Paradise	5–14	10 min/8 episodes	Immersive experience visiting Chimelong Safari Park, Ocean Kingdom, Water Park, Happy World, Bird Paradise, International Circus and other attractions.
Nature Real Scene	Exploring the Fantasy of the Sea	5–14	10 min	Tranquil and healing underwater scenery featuring coral reefs and colorful fish swimming freely. Accompanied by bubble sounds and fish movement sounds, creating an immersive sense of serenity.
Animation Paradise	Adventure in Eggy World	5–8	5 min/3 episodes	Eggy characters may pass through glowing rainbow arches into a sky kingdom full of flying cotton candy clouds, or stumble into a sweet town built of candies, or venture into a mysterious magic forest.
Animation Paradise	The Campus Life of a Mischievous Kid	5–8	15 min	The story centers on a quirky boy named “Guai Doudou,” who fancies himself both a “math whiz” and a “junior detective”. It recounts the humorous and delightful adventures of his everyday life.
Wonderful Adventure	Journey Through Ancient Texts: Juyan Han Bamboo Slips	8–14	15 min	Become a frontier soldier, climb the Great Wall, look out from the beacon tower, and experience life guarding the border during the Eastern Han Dynasty, protecting national security.

**Table 2:** General data between two groups.

Variables	Intervention Group (*n* = 40)	Control Group (*n* = 40)	*p*-Value (<0.05) *
Age (years), Mean (SD)	8.05 ± 2.60	8.63 ± 2.50	0.316 ^a^
Gender, n (%)			0.491 ^b^
Male	26 (65.0)	23 (57.5)	
Female	14 (35.0)	17 (42.5)	
Height (cm), Median (IQR)	128 (120.25, 140)	130 (123.5, 140)	0.383 ^c^
Weight (kg), Median (IQR)	24.5 (20.9, 33.7)	26 (24, 32.7)	0.133 ^c^
Diagnosis, n (%)			0.896 ^d^
Respiratory System Disease	26 (65.0)	27 (67.5)	
Digestive System Disease	1 (2.5)	1 (2.5)	
Short Stature	10 (25.0)	7 (17.5)	
Fever	2 (5.0)	3 (7.5)	
Other	1 (2.5)	2 (5.0)	
VR Experience, n (%)			0.809 ^b^
Yes	13 (32.5)	12 (30.0)	
No	27 (67.5)	28 (70.0)	
Hospital stays in past, n (%)			0.183 ^b^
0	17 (42.5)	10 (25.0)	
1–2	17 (42.5)	19 (47.5)	
≥3	6 (15.0)	11 (27.5)	
Number of Peripheral Venous, n (%) Catheterizations			0.259 ^b^
0	10 (25.0)	8 (20.0)	
1	10 (25.0)	5 (12.5)	
2–5	8 (20.0)	15 (37.5)	
≥6	12 (30.0)	12 (30.0)	
Primary Caregiver, n (%)			0.742 ^d^
Mother	29 (72.5)	27 (67.5)	
Father	8 (20.0)	11 (27.5)	
Grandparents	3 (7.5)	2 (5.0)	
Caregiver’s Education Level, n (%)			0.481 ^d^
High School or Below	6 (15.0)	3 (7.5)	
College (Associate Degree) or Above	34 (85.0)	37 (92.5)	

Note: * *p* < 0.05 is significant; ^a^ *t*-test; ^b^ Pearson’s chi-square test; ^c^ Mann–Whitney U test; ^d^ Fishers exact test.

**Table 3:** Outcome measures between the control group and the intervention group at different time points.

Variables	Control Group (n = 40)	Intervention Group (n = 40)	*p*-Value (<0.05) *	Effect Size (95%CI)
WB-FPRS pain score Median (IQR)				
T0	0 (0,0)	0 (0, 0)	1.000 ^c^	0.00 (0.00, 0.00) ^e^
T1	4 (2.5, 5)	2 (1, 4)	0.029 ^c^	0.28 (0.02, 0.50) ^e^
T2	0.5 (0, 2)	0 (0, 2)	0.037 ^c^	0.24 (0.02, 0.46) ^e^
VAS-A anxiety score Median (IQR)				
T0	5 (2.25, 7)	5 (3, 7)	0.665 ^c^	0.06 (−0.19, 0.31) ^e^
T1	1 (0, 3)	0 (0, 2)	0.038 ^c^	0.25 (0.01, 0.47) ^e^
T2	0 (0, 1)	0 (0, 0)	0.011 ^c^	0.25 (0.06, 0.43) ^e^
Heart Rate (beats/min), Mean (SD)				
T0	104.07 ± 2.86	103.82 ± 2.98	0.829 ^a^	0.01 (−0.43, 0.45) ^f^
Tmax	115.25 ± 29.53	107.67 ± 16.25	0.047 ^a^	0.54 (0.09, 0.98) ^f^
T2	101.76 ± 16.24	99.63 ± 17.18	0.559 ^a^	0.35 (−0.09, 0.79) ^f^
Procedure time, (seconds), Median (IQR)	120 (110, 123.5)	110 (100, 120)	<0.001 ^c^	0.44 (0.23, 0.62) ^e^

Note: * *p* < 0.05 is significant; ^a^ *t*-test; ^c^ Mann–Whitney U test; ^e^ Rank-biserial r effect size; ^f^ Cohen’s d effect size.

**Table 4:** Comprehensive Results of W-BFPS Generalized Estimating Equations (GEE) Parameter Estimation and Effect Testing.

Parameter	Regression Coefficient (β)	SE	95% Wald CI	Wald (2)	*df*	*p*
nodal increment	1.40	0.28	0.84–1.96	24.20	1	<0.001
Grouping (Experimental vs. Control)	−0.78	0.32	−1.40–(−0.15)	5.90	1	0.015
Measurement time (T0 vs. T2)	−1.40	0.28	−1.96–(−0.84)	24.20	1	<0.001
Measurement time (T1 vs. T2)	2.38	0.36	1.67–3.08	43.52	1	<0.001
Experimental group (vs control group) × Δ(T0-T2) (interaction term)	0.78	0.32	0.15–1.40	5.90	1	0.015
Experimental group (vs control group) × Δ(T1-T2) (interaction term)	−0.45	0.46	−1.36–0.46	0.94	1	0.332

Note: The dependent variable is W-BFPS, with a QIC of 531.850, indicating good model fit; *p* < 0.05 signifies statistically significant differences.

**Table 5:** VAS-A Generalized Estimating Equations (GEE) Parameter Estimation and Effectiveness Testing Comprehensive Results.

Parameter	Regression Coefficient (β)	SE	95% Wald CI	Wald (2)	*df*	*p*
nodal increment	0.60	0.16	0.29~0.91	14.55	1	<0.001
Grouping (Experimental vs. Control)	−0.43	0.18	−0.77~−0.08	5.85	1	0.016
Measurement time (T0 vs. T2)	4.28	0.49	3.31~5.24	75.37	1	<0.001
Measurement time (T1 vs. T2)	1.28	0.32	0.64~1.91	15.48	1	<0.001
Experimental group (vs control group) × Δ(T0-T2) (interaction term)	0.25	0.65	−1.02~1.52	0.15	1	0.700
Experimental group (vs control group) × Δ(T1-T2) (interaction term)	−0.40	0.39	−1.17~0.37	1.04	1	0.308

Note: The dependent variable was VAS-A, with a QIC of 1074.425, indicating good model fit; *p* < 0.05 was considered statistically significant.

**Table 6:** Patient Satisfaction with Nursing Procedure.

Variable	Control Group (n = 40)	Intervention Group (n = 40)	*p*-Value (*p* < 0.05)
Operator Satisfaction, n (%)			
Overall Satisfaction (Combined)	29 (72.5)	38 (95.0)	<0.001
Very Satisfied	8 (20.0)	30 (75.0)	
Satisfied	21 (52.5)	8 (20.0)	
Moderately Satisfied	11 (27.5)	2 (5.0)	

Note: Comparison of overall satisfaction between the two groups: χ^2^ = 24.795, *p* < 0.001.

**Table 7:** VR Headset Usability Evaluation.

Measurement Indicator	Level	Number of Cases, n (%)
Comfort of VR Headset	Very comfortable	13 (32.5)
	Comfortable	25 (62.5)
	Somewhat comfortable	1 (2.5)
	Somewhat uncomfortable	1 (2.5)
	Uncomfortable	0 (0.0)
Fatigue Sensation	Not fatigued at all	36 (90.0)
	A little fatigued	4 (10.0)
	Moderately fatigued	0 (0.0)
	Very fatigued	0 (0.0)
Usage Score	8–10 points	36 (90.0)
	5–7 points	3(7.5)
	<5 points	1 (2.5)
Nausea/Dizziness Sensation	None	38 (95.0)
	Present	2 (5.0)
Willingness	Willing	38 (95.0)
	Neutral	1 (2.5)
	Unwilling	1 (2.5)

Note: Data are presented as number of cases and percentage. The percentage for “5–7 points” has been adjusted to 7.5% based on the total sample size (n = 40).

## Data Availability

The datasets generated during and/or analyzed during the current study, including de-identified individual participant data and the data dictionary, are available from the corresponding author upon reasonable request.

## Abbreviations

The following abbreviations are used in this manuscript:

VR	V irtual R eality
RCT	R andomized C ontrolled T rial
WB-FPRS	Wong–Baker Faces Pain Rating Scale
VAS-A	Visual Analog Scale for Anxiety
GEE	Generalized Estimating Equations
